# Whole Blood, Fixed Ratio, or Goal-Directed Blood Component Therapy for the Initial Resuscitation of Severely Hemorrhaging Trauma Patients: A Narrative Review

**DOI:** 10.3390/jcm10020320

**Published:** 2021-01-17

**Authors:** Mark Walsh, Ernest E. Moore, Hunter B. Moore, Scott Thomas, Hau C. Kwaan, Jacob Speybroeck, Mathew Marsee, Connor M. Bunch, John Stillson, Anthony V. Thomas, Annie Grisoli, John Aversa, Daniel Fulkerson, Stefani Vande Lune, Lucas Sjeklocha, Quincy K. Tran

**Affiliations:** 1 Notre Dame Campus, Indiana University School of Medicine, South Bend, IN 46617, USA; markwalshmd@gmail.com (M.W.); speybroeckjacob@gmail.com (J.S.); mkmarsee@gmail.com (M.M.); cmbunch@iu.edu (C.M.B.); jstills@iu.edu (J.S.); 33thomasa@gmail.com (A.V.T.); agrisoli@iu.edu (A.G.); 2 Departments of Emergency & Internal Medicine, Saint Joseph Regional Medical Center, Mishawaka, IN 46545, USA; 3 Ernest E. Moore Shock Trauma Center, Denver Health, Denver, CO 80204, USA; Ernest.moore@dhha.org; 4 Department of Surgery, University of Colorado Health Science Center, Denver, CO 80204, USA; hunter.moore@ucdenver.edu; 5 Department of Trauma Surgery, Memorial Leighton Trauma Center, Beacon Health System, South Bend, IN 46601, USA; SThomas@beaconhealthsystem.org; 6 Division of Hematology and Oncology, Department of Medicine, Northwestern University Feinberg School of Medicine, Chicago, IL 60611, USA; h-kwaan@northwestern.edu; 7 Department of Surgery, Indiana University School of Medicine, Indianapolis, IN 46202, USA; javersa@iupui.edu; 8 Department of Neurosurgery, Beacon Medical Group, South Bend, IN 46601, USA; dhfulkerson@beaconhealthsystem.org; 9 Emergency Medicine Department, Navy Medicine Readiness and Training Command, Portsmouth, VA 23708, USA; stefani.vandelune@gmail.com; 10 The R Adams Cowley Shock Trauma Center, University of Maryland School of Medicine, Baltimore, MD 21201, USA; lsjeklocha@som.umaryland.edu

**Keywords:** blood transfusion, blood component transfusion, exsanguination, fibrinogen, hemostatics, thromboelastography

## Abstract

This narrative review explores the pathophysiology, geographic variation, and historical developments underlying the selection of fixed ratio versus whole blood resuscitation for hemorrhaging trauma patients. We also detail a physiologically driven and goal-directed alternative to fixed ratio and whole blood, whereby viscoelastic testing guides the administration of blood components and factor concentrates to the severely bleeding trauma patient. The major studies of each resuscitation method are highlighted, and upcoming comparative trials are detailed.

## 1. Introduction: Rationale for the Adoption of Whole Blood and Fixed Ratio Resuscitation

### 1.1. History of the United States’ Swift Adoption of Cold-Stored Whole Blood for Civilian Urban Trauma Resuscitation

Since 2016, many large United States academic centers have embarked upon an ambitious program to offer whole blood (WB) in the civilian and urban prehospital environment for severely hemorrhaging patients. This process required variances from the American Association of Blood Banks recommendations [1,2]. In austere and rural environments where no blood banking or testing is available, cold-stored WB (CSWB) may be the best empiric agent for prehospital resuscitation of the severely bleeding patient. Prehospital fresh frozen plasma is commonly available. However, recent studies demonstrate that platelet dysfunction and fibrinogen deficiency are of paramount importance in early trauma-induced coagulopathy (TIC) [1,2,3,4]. Therefore, some urban academic trauma systems with short transport times have been early adopters of CSWB. Many of these academic centers who advocate for trauma resuscitation with WB are led by trauma specialists with military backgrounds [1,2]. In turn, there has been a significant drive to provide CSWB not only in the rural civilian environment—where its use is beneficial given transport times and resource limitations—but also to the urban nonacademic environment with short transport times [1,2,3,4,5,6,7,8,9,10]. Without access to similar robust academic and/or military ties, these nonacademic trauma centers may not have the resources to offer WB as easily as their academic and military counterparts. Therefore, the mechanistic rationale for the provision of CSWB in the civilian and urban environment requires historical evaluation prior to its widespread adoption.

### 1.2. PROPPR Trial as Mechanistic Rationale for Justification of CSWB

WB resuscitation garnered practical support by the demonstration that a fixed ratio of equal parts plasma, platelets (PLTs), and packed red blood cells (PRBCs) improves mortality. The 2015 Pragmatic, Randomized Optimal Platelet and Plasma Ratios (PROPPR) trial established use of balanced blood transfusion. This study of 680 patients from 12 centers suggested that a fixed ratio of 1:1:1 (plasma/PLTs/PRBC) provided a benefit to mortality caused by exsanguination at 24 h but no overall mortality benefit when compared to a ratio of 1:1:2 [11]. The PROPPR trial has received significant scrutiny because of the lack of benefit in overall 24 h mortality. The trial has also received criticism for inconsistencies and confounding variables. PLTs were not provided to the 1:1:2 group until after 6 units of PRBCs were administered. Moreover, cryoprecipitate or fibrinogen were not components of either regimen. Plasma was often administered well after the initial resuscitation period but within the first 24 h. This phenomenon was termed “catch up” and implied that these patients did not receive equal components during the critical first few hours of resuscitation. Therefore, patients never truly received a fixed 1:1:1 ratio during the initial resuscitation period. Finally, patients whose cause of death was exsanguination included those who also had traumatic brain injury (TBI), which confounded the cause of death [11,12,13,14]. A follow-up systematic review of 16 randomized controlled trials (RCTs) stated similar concerns regarding the recommendations of the PROPPR trial. Given the diversity of patients with blunt or penetrating trauma and with or without TBI, additional studies are required to determine the mortality benefit of 1:1:1 vs. 1:1:2 fixed ratios [12,15,16]. Due to the concerns about the validity of the PROPPR trial, which set the standard for the fixed 1:1:1 practice, we review the reasons for the widespread adoption of CSWB in the United States for civilian urban trauma.

### 1.3. Historical Justification for CSWB in Civilian Urban Trauma 

Few retrospective studies and only one RCT compared WB versus fixed ratio for resuscitation in the urban civilian population (Table 1). The first study comparing WB versus standard blood component therapy (BCT) for civilian resuscitation was published in 2009. Since then, the paucity of literature mostly constitutes editorials or papers on the feasibility of applying far forward combat WB use to the civilian arena. A total of 1382 civilian patients form the foundation of WB’s widespread adoption in the United States’ urban areas [1,2,3,4,5,6,17,18,19,20,21,22,23,24,25,26,27,28,29,30,31,32,33,34,35,36,37,38]. The most recent small studies revealed an equivocal mortality benefit from overuse of WB because “it was easier and faster in a chaotic and busy trauma bay” [29]. Moreover, it was recently noted that “WB is not effective at treating traumatic hemorrhage in resuscitation as compared with component therapy” [33]. Broad introduction of CSWB in the absence of high-quality trials will likely lead to further heterogeneity of guidelines among hospital systems and education programs. Three RCTs, discussed later, are currently underway and the results will not be reported for over a year. Given the scant literature justifying CSWB in the prehospital and urban environment for trauma, and the concerns regarding the PROPPR trial, consideration of viscoelastic test (VET)-guided trauma resuscitation is warranted. 

## 2. VET-Guided Goal-Directed Resuscitation

The rationale of CSWB and fixed ratios is based on the principle that the patient should be “given what they have lost” [39]. There are significant temporal changes within the complex spectrum of TIC within the first hours of resuscitation [40]. The complex pathophysiology of TIC often persists after the simple administration of fixed ratios or WB, and TIC remains a leading cause of mortality after resuscitation. TIC phenotypes, an intricate web of hemostatic and immunologic aberrancies, depend on factors such as penetrating versus blunt trauma, the presence of TBI, the time from injury to resuscitation, the genetic hematologic makeup of the patient, and the methods of resuscitation. The administration of either fixed ratios or WB does not represent a precision-based therapeutic approach to the individual patient’s hemostatic derangements in TIC, especially when guided only by common coagulations tests such as the prothrombin time, international normalized ratio, activated partial thromboplastin time, PLT count, and fibrinogen. As a result, there has been advocacy for administration of BCT under the guidance of the VETs, including thromboelastography (TEG) and rotational thromboelastometry (ROTEM) [41,42,43,44,45,46,47,48,49,50,51,52,53,54,55,56]. This strategy of VET-guided goal-directed BCT (GD BCT) can be considered a better alternative for individualized resuscitation of the hemorrhaging trauma patient. 

### 2.1. History of VET and Trauma Resuscitation

In 1997, the first case series of 67 trauma patients whose BCT was guided by TEG was published in the United States [57]. Since then, numerous papers in worldwide journals have detailed how TEG/ROTEM provides more efficient resuscitation of severe bleeding in not only trauma, but also surgical anesthesia and obstetrics [42,58,59,60]. However, the adoption of the TEG/ROTEM in the United States has remained stagnant. In 2016, it was discovered that only 9% of institutions used VET-guided BCT for patients who required large volume resuscitation [61,62]. Meanwhile, our European colleagues have adopted early and repeated VET-guided BCT as the bedrock of modern trauma resuscitation. Since the beginning of the century, European guidelines and their iterations have recommended VET-guided BCT for initial and ongoing resuscitation of the severely bleeding trauma patient [63,64,65]. Despite this significant history in Europe, VET-guided BCT has failed to gain acceptance in the United States where continued emphasis on common coagulations tests guide therapy. This may be explained in part by the inherent bias of blood bankers and hematologists who often lack familiarity with TEG/ROTEM due to institutional variability caused often by pipetting inconsistencies [66,67]. Standardization of VETs is being addressed with controlled cartridge systems, such as the TEG6S and ROTEM Sigma. These enable easier implementation of rapid test variants of VET assays and decreased time to potentially actionable information [68,69,70,71]. 

Initial trauma resuscitation in the United States may be led by either a trauma surgeon and anesthesiologist, or an emergency physician. There has been a recent push in the United States to approach trauma care with more attention to the pathophysiology of TIC. Thus, it is vital that acute care trauma surgeons and critical care surgeons are provided the educational impetus to advance the use of the VETs seamlessly throughout the hospital. Whether in Europe or the United States, a seamless transitioning of TIC prevention and management—guided by TEG/ROTEM—has resulted in a more physiologic replenishment of blood products in the trauma patient [68,69,70,72,73].

Since 2012, Germany has undergone concerted educational and institutional efforts to use VETs to guide trauma resuscitation in a more physiologic manner. At the beginning of the century, the multicenter trauma registry of the German Society for Trauma analyzed ratios of plasma, PRBCs, and PLTs and administration of coagulation factor concentrates (CFCs) in adult trauma. As a result, a significant reduction in crystalloid use and a higher ratio of plasma and PLTs to PRBC use, as well as an increase in CFCs, were noted to improve mortality in the studied time frame. Most recently, the findings of the German study group have advanced many areas of VET-guided BCT and hemostatic adjunct therapy with 4-factor prothrombin complex concentrate (PCC) and soluble fibrinogen [47,74].

A Copenhagen group used a hybrid approach of an initial 1:1:1 fixed ratio followed by TEG-guided GD BCT with factor concentrates. This landmark study published in 2010 establishes the “Copenhagen Concept”, which has become a standard for the treatment of hemorrhaging trauma patients [54,75]. Likewise, an Italian group using ROTEM-guided therapy has demonstrated a similar protocol wherein a preemptive dose of 2 g fibrinogen concentrate is administered for trauma patients in hemorrhagic shock. The Italian group demonstrated reduced blood product consumption, decreased overall cost, and improved early and 28 day mortality [76].

### 2.2. History of VET Goal-Directed Therapy with Coagulation Factor Concentrate 

Personalized VET-guided direction of BCT possesses theoretical advantages over the WB and ratio-driven methods. Despite the increasing availability of rapidly obtainable VET results, which allow for the targeted supplementation of procoagulants, consensus among physicians regarding the use of factor concentrates is lacking, particularly in the United States. Prospective studies and RCTs comparing GD BCT with and without CFCs are necessary

Not all blood components are depleted equally in hemorrhage. Resuscitation itself causes the dilution of the coagulation process and factors. There has been renewed interest for the use of VET-guided fibrinogen administration in Europe and the United States. Fibrinogen is the precursor for clot formation and its level determines the clot stability early in resuscitation. Early prehospital and emergency department fibrinogen for preventing the progression of TIC has been a significant area of recent study. Attention has also been brought to early hospital use of PLT therapy in order to enhance the stability of the fibrin/PLT plug as studies have shown that higher ratios of PLTs provided early to patients with surgical and traumatic bleeding are associated with increased survival [77,78,79,80,81,82,83,84].

European studies reveal that cryoprecipitate replacement for fibrinogen levels greater than 1–5 g/L was feasible in patients who required massive transfusion (MT). Studies have demonstrated the feasibility of providing fibrinogen concentrate to severely bleeding trauma patients. The Fibrinogen in the initial Resuscitation of Severe Trauma (FiiRST), REversal of Trauma-Induced Coagulopathy (RETIC), and Early-Fibrinogen In Trauma 1 (E-FIT1) studies, as well as the European prehospital Fibrinogen in Trauma-Induced Coagulopathy (FinTIC) trial, have demonstrated that soluble fibrinogen given early during trauma resuscitation may result in significant clinical benefit. The RETIC study used plasma in the control arm to treat early TIC and compared it to soluble fibrinogen in patients in whom ROTEM indicated a fibrinogen deficiency. The study was terminated early because a significant number in the plasma group required rescue therapy and MT compared to the CFC group. Soluble fibrinogen is costly while cryoprecipitate is less expensive and more readily available. The CRYOSTAT1 and two trials are designed to study the efficacy of cryoprecipitate for the maintenance of resuscitation for fibrinogen levels greater than 1.8 g/L. These trials advocate for the early administration of cryoprecipitate in the prevention and treatment of early TIC because of the early fibrinogen derangement as a cause of incipient TIC. Recent studies indicate that timely soluble fibrinogen concentrate administration is possible in the prehospital and urban hospital environment, with ROTEM evidence of increased clot stability. Future studies have been proposed to provide soluble fibrinogen and 4-factor PCC in the prehospital and hospital civilian urban setting as an alternative to fixed ratio or WB resuscitation [85,86,87,88,89,90,91,92,93,94,95,96]. Since 2017 in Australia, the Fibrinogen Early In Severe Trauma study (FEISTY) is a RCT attempting to address the validity of soluble fibrinogen for early resuscitation of severely bleeding trauma patients [97]. 

With precision-based medicine guided by VETs, the hemorrhaging trauma patient’s resuscitation is provided by a finely-tuned menu to address their individual hemostatic deficits. TEG/ROTEM-guided therapies represent an area of promise mastered by more than a few European centers, but lags in the United States where CFCs are not yet approved for trauma resuscitation [98].

### 2.3. Recent Challenge to VET-Guided Trauma Resuscitation: The iTACTIC Trial

A recent RCT has challenged the value of VET-guided BCT and CFC in severely bleeding patients. The implementing Treatment Algorithms for the Correction of Trauma-Induced Coagulopathy (iTACTIC) trial compared VETs to the standard coagulation tests in bleeding trauma patients. This trial demonstrated no statistical evidence that the use of VETs versus standard therapy guided by standard coagulation tests improved 28 day mortality. However, similar confounding factors were also found with the PROPPR trial [99]. Despite the high injury severity scores, a relatively small percentage of either group received MT within 24 h. This is also reflected by the low incidence of TIC in both groups. Like the PROPPR trial, no proportion of patients were identified with significant surgical bleeding. Moreover, there was a confluence of those patients with TBI and hemorrhagic death, rendering any causal link of death to hemorrhage problematic. While some of the centers in the iTACTIC trial had been performing TEG and ROTEM at a clinical level for many years, other centers began their practical experience with VETs at the beginning of the study. The authors of the iTACTIC study note the challenges in attempted performance homogeneity among the seven centers. This raises significant concern for the validity of the results, as there is a steep learning curve in the adoption of VETs to guide BCT and CFCs for severely bleeding patients. Additionally, the authors of the trial do not comment on the percentage of centers which used either ROTEM or TEG devices to guide BCT. They state: “The [VET] used at each study site was determined by existing familiarity with a specific device appliance and to ensure a balanced use of the devices across the study” [99]. This is of importance due to the heterogeneity of thresholds for administering BCT [100,101]. Finally, a problem consistent with many studies regarding the treatment and diagnosis of patients with severe bleeding and trauma is the definition of MT as 10 units PRBCs in 24 h. This may explain the low incidence of TIC in the iTACTIC study considering that the most effective time for intervention to reverse the pathophysiology of TIC is the first few hours after injury. Only 28% in the standard coagulation tests group and 26% in the VET group had received MT at 24 h [99]. This traditional MT definition restricts the potential study sample to a less acute group of patients, excludes patients who die early, and excludes those who may have benefitted from early use of VET-guided resuscitation [102]. These issues indicate that the population studied may not have been sick enough to benefit from VET-guided trauma resuscitation [99]. More recent data suggest that >4 units PRBCs in the first hour is a more meaningful definition of MT [103].

## 3. Geographic Variations

Comparison of the United States and European trauma data is confounded by the relatively high incidence of penetrating injury in the United States. The United States’ emphasis on WB may stem from this high incidence of penetrating injury as an early cause of potentially preventable hemorrhage (PPH), serving as an impetus to provide WB in the civilian urban environment. It has been reported that 34.5% of patients died from PPH in the prehospital setting or within an hour of hospitalization for all forms of trauma. Hence, the use of WB in the early prehospital and emergency department environment has been viewed as a more effective method for prehospital resuscitation to decrease deaths from PPH [104]. 

The incidence of penetrating injury varies among developed countries. In major United States trauma centers in large metropolitan areas, the incidence may be as high as 45% of all injuries, whereas in Europe the percentage of penetrating injuries is much lower (0.2–5% in Switzerland, the Netherlands, and Germany) [105,106].

Resuscitation of these penetrating injuries also depends on immediate surgical control of hemostasis. Therefore, this population may not be directly comparable to the European population, where motor vehicle accidents with a high incidence of TBI confer different methodologies of resuscitation. Nevertheless, it is notable that the country with the highest use of VETs also has the least use of prehospital blood products administered in a fixed ratio. Germany, with its centralized system and relatively short transport times, uses prehospital blood products in 6% of trauma centers. Comparatively in France, the rate is 89% [16]. It is not surprising that the only European RCT for using CSWB in the civilian urban population is in France, where the time spent in the field on resuscitation efforts is longer than the “scoop and run” strategy used in the United States, where physicians are not onboard ambulances [107].

The equivocal success of prehospital administration of blood products in the civilian urban environment has been inconsistently replicated in other studies. A recent review of the European literature suggests that prehospital blood transfusion with or without PRBCs resulted in equivocal reduction of 24 h mortality [15,85,108]. The authors caution that “based on mainly poor-quality evidence, no hard conclusion can be drawn about a possible survival benefit for hemorrhagic trauma patients receiving prehospital blood transfusion. Overall, prehospital blood transfusion is safe, but the results of currently ongoing RCTs await to demonstrate a survival benefit.” [15]. However, the European Society of Anesthesiology recently reported that 48% of responders to an online survey had access to PRBCs, 22% to fresh plasma, and 14% to lyophilized plasma [16]. It is clear that there were significant dissimilarities in practice among European countries with no consensus regarding the benefit of prehospital blood products [16]. Hence, there is a tremendous need for continued investigation in RCTs. 

On the other hand, European trauma specialists have provided substantial literature confirming the use of VETs to guide resuscitation in the last two decades. An expanding emphasis for use of VET-guided BCT in trauma resuscitation has developed throughout Europe and the United States [40,42,44,46,47,54,55,90,94,109,110,111,112,113,114,115,116,117,118,119,120,121].

The most significant difference between Europe and the United States concerns the frequent use of VET-guided administration of PCC, soluble fibrinogen, PRBCs, and PLTs. The European reliance on VET-guided resuscitation and CFC therapy is significant; 2019 guidelines no longer recommend the use of plasma for the treatment of hypofibrinogenemia.

The European guidelines are significant in that fibrinogen and PLT dysfunction are addressed among the first abnormalities in patients with TIC. The volume of plasma required to correct this fibrin deficiency, documented by ROTEM, would result in unnecessary dilution and potential worsening of the coagulopathy. The RETIC study demonstrated the superiority of CFC when compared to plasma [78,79,80,81,82,83,84]. Highly specialized trauma centers in Switzerland, Austria, and Germany have published studies demonstrating mortality improvement for patients with severe trauma who are treated in a goal-directed fashion with CFCs and minimal crystalloid [78,85,87,94,111,118,122,123].

VET-guided GD BCT has now been used in the United States and Europe with increasing frequency and earlier in the phases of care. From our literature search, there was no evidence of VET use in the prehospital environment. VETs are not useful in the prehospital setting because vibrations in a moving ambulance result in an inaccurate tracing.

## 4. Future Direction—RCTs in Progress

There are three ongoing trials for early trauma resuscitation with WB for severely bleeding patients, the PPOWER trial, the STORHM trial, and the SWAT trial. 

### 4.1. PPOWER

The Pragmatic, Prehospital, Type O, Whole Blood Early Resuscitation (PPOWER) trial, a single-center, 3 year, prospective randomized pilot trial of type O low-titer, leukocyte-reduced (LTLR) WB, is in progress at Pittsburgh, United States. This trial is based in the helicopter prehospital setting followed by LTLR WB in the hospital. Four helicopter centers are provided with WB for this study. Patients will receive 6 units of WB upon entry into the study. Of note, previous protocols called for 2 units of WB, then transitioned to TEG-guided GD BCT. This study provides a higher number of WB units followed by TEG-guided resuscitation, rendering this a hybrid study combining the use of WB, fixed ratios, and TEG-guided GD BCT. The hypothesis of this study is that WB will provide better outcomes for the early resuscitation of trauma patients, particularly when combined with TEG-guided BCT. Proposed completion is by late 2021 [124].

### 4.2. STORHM

A French study (Sang Total pour la Reanimation des Hemorragies Massives, STORHM) is still in the planning stage as a noninferiority study to compare LTLR WB to a 1:1:1 fixed ratio for the severely hemorrhaging trauma patient. Like the PPOWER study, the endpoint will be mortality, but TEG parameters will also be analyzed. Other endpoints are to include lactate clearance and the presence of multiorgan failure at 24 h. It is anticipated that this trial, which began in late 2019, will recruit 200 patients from six trauma centers [125].

### 4.3. SWAT

Finally, the Linking Investigations in Trauma and Emergency Services network—a consortium of trauma centers that conduct prospective, multicenter, injury care, and outcomes research—has sponsored the Shock, Whole Blood, and Assessment of TBI (SWAT) trial to compare WB and standard BCT. This trial is a 4 year, multicenter, prospective, observational cohort study that will analyze early WB resuscitation compared to standard BCT resuscitation for trauma patients with severe hemorrhagic shock, with and without TBI. Completion is scheduled for February 2022 [9].

## 5. Conclusion

Historical, geographical, and mechanistic components influence the selection of fixed ratio blood component and WB resuscitation for severely bleeding trauma patients. Because of an institutional reliance in the United States, based on the paradigm of far forward combat resuscitation where WB is commonly used, a rapid implementation of WB in the civilian urban resuscitation has occurred. In much of Europe, however, with a long tradition of VET-guided trauma resuscitation, fixed ratio resuscitation is less commonly used and WB is only used in the initial stages of investigation. Until recently, few academic trauma centers in the United States have used VETs to guide trauma resuscitation, rather adhering to fixed ratios or CSWB. The advantages and disadvantages of the three resuscitation methods are summarized in Table 2.

## Figures and Tables

**Table 1:** Studies of WB in the Civilian Population.

Study	Number of Patients Receiving WB	Study Design	Description	Results
Cotton et al. [28]	55	RCT	Single-center, RCT comparing modified WB and component therapy	No difference in 30 day mortality. Possible transfusion benefit in TBI.
Williams et al. [33]	198	Comparative clinical prospective therapeutic study	Comparison of LTOWB versus BCT in prehospital and ED setting for trauma patients	LTOWB received less post-ED blood products with equivocally mortality benefit.
Hanna et al. [36]	280	Retrospective cohort analysis	Analysis of 2015–2016 ACS TQIP database comparing patients in hemorrhagic shock and given at least one unit of PRBCs or WB (note this likely includes patients from other studies)	WB associated with improved 24 h mortality overall and improved in-hospital mortality in subgroups with penetrating mechanism and those without severe head injury.
Ho and Leonard [31]	77	Retrospective cohort study	Unrefrigerated young WB transfusion for patients requiring massive transfusion in a civilian setting	No benefit (overall survival and transfusion were equivalent)
Leeper et al. [38]	28	Propensity matched cohort	Propensity matched cohort of children over 1 year	No difference in mortality. Improved indices of shock and coagulopathy.
Seheult et al. [22]	172	Retrospective case–control analysis	Safety analysis of LTOWB patients by blood group	No mortality difference, similar clinical outcomes across groups, no increased hemolysis noted at 24 h in nongroup O patients.
Fadeyi et al. [35]	167	Retrospective cohort	Comparison trauma patient receiving LTOWB with leuko-reduced LTOWB	Nonstatistically significant 6% increased mortality in leuko-reduced LTOWB (*p* = 0.185).
Hazelton et al. [30]	107	Dual-center case-match study	Matching analysis for patients who received CSWB vs. BCT for hemodynamic parameters, hemoglobin and hematocrit at 24 h, trauma bay mortality, and 30 day mortality	CSWB associated with improved trauma bay survival and higher mean hemoglobin value at 24 h. No difference in the amount of blood products transfused at 4 and 24 h periods.No difference in 30 day mortality between the two groups.
Shea et al. [37]	44	Prospective observational	Before-and-after comparison of trauma patient requiring activation of massive transfusion before and after implementation of LTOWB MTP (up to 8 LTOWB unit)	No difference of absolute mortality between groups. Post-hoc multivariate regression showed improved adjusted mortality in LTOWB group. Authors argue for effect mediation based on ROTEM parameters.
Gallaher et al. [29]	42	Retrospective observational	Before-and-after comparison of mortality in trauma patients receiving blood products during implementation of LTOWB program	LTOWB did not alter 30 day mortality, nonstatistically significant increase in total blood products, and no difference in lab values at 48 h.
Zielinski et al. [2]	24 Mayo 22 Pittsburgh 73 Royal Caribbean	Retrospective observational	Describes the THOR network prehospital WB programs including Mayo Clinic and Allegheny General Hospital and WFWB transfusion on Royal Caribbean cruise	Observation only. Overall mortality in patients who received WB 31%.
Seheult et al. [25]	44	Observational study	Hemolysis markers in group O and nongroup O recipients of platelet replete, uncrossmatched CSWB	No difference in hemolysis markers across groups.
Zhu et al. [19]	30	Retrospective observational	Descriptive study of prehospital WB transfusion implementation which included transfusion of 25 adults and 5 pediatric patients	Observational. Mortality in adult: 36%; mean ISS: 29. Mortality in pediatric: 20%; mean ISS: 29. Mean transport time, 37 min.
Leeper et al. [20]	18	Retrospective observational	Pediatric uncrossmatched LTOWB transfusion for hemorrhagic shock	ISS: 34. Mortality 44%.
Condron et al. [27]	1	Case report	Case report of massive transfusion using 38 units of LTOWB in addition to MTP with blood components	N/A

Abbreviations: ACS, American College of Surgeons; BCT, blood component therapy; CSWB, cold-stored whole blood; ED, emergency department; ISS, injury severity score; LTOWB, low-titer group O whole blood; MTP, massive transfusion protocol; PRBCs, packed red blood cells; RCT, randomized controlled trial; ROTEM, rotational thromboelastometry; TBI, traumatic brain injury; THOR, Trauma Hemostasis and Oxygenation Research; TQIP, Trauma Quality Improvement Program; WB, whole blood; WFWB, warm fresh whole blood.

**Table 2:** Advantages and disadvantages of whole blood vs. fixed ratio vs. VET-guided BCT.

	Advantages	Disadvantages
Whole blood	- Simple protocols and uniform dosing - Easy to administer in prehospital and hospital setting - Provides all blood components; avoids hemodilution	- Not physiologically driven - Few RCTs - Unnecessary blood components may be given- Products currently not universally available; shorter shelf life - Requires AABB variances and additional cost
Fixed ratio	- Simple protocols and uniform dosing - Easy to administer in prehospital and hospital setting - More RCTs, but questionable validity regarding 1:1:1 vs. 1:1:2 - Blood components readily available	- Not physiologically driven - Unnecessary blood components may be given - Does not replenish fibrinogen effectively in early stages
VET-guided BCT	- Provides early physiologically driven resuscitation - Limits blood product waste - Blood components readily available	- Protocols not straight forward; accuracy operator dependent; steep learning curve - Cannot be used in prehospital setting - Few RCTs

Abbreviations: AABB, American Association of Blood Banks; BCT, blood component therapy; RCT, randomized control trial; VET, viscoelastic test [1,2,3,4,5,6,7,8,9,10,11,14,40,42,46,47,52,54,59,61,69,78,85,86,87,89,94,95,98,99,100,109,115,126].

## Data Availability

This study did not report any data.

## Abbreviations

AABB	American Association of Blood Banks
BCT	blood component therapy
CFC	coagulation factor concentrates
CSWB	cold-stored whole blood
E-FIT1	Early-Fibrinogen In Trauma 1
FEISTY	Fibrinogen Early In Severe Trauma study
FiiRST	Fibrinogen in the initial Resuscitation of Severe Trauma
FinTIC	European prehospital Fibrinogen in Trauma-Induced Coagulopathy
GD BCT	goal-directed blood component therapy
ISS	injury severity score
iTACTIC	Treatment Algorithms for the Correction of Trauma-Induced Coagulopathy
LTLR	low-titer, leukocyte-reduced
LTOWB	low-titer group O whole blood
MT	massive transfusion
MTP	massive transfusion protocol
PCC	prothrombin complex concentrate
PLT	platelet
PPH	potentially preventable hemorrhage
PPOWER	Pragmatic, Prehospital, Type O, Whole Blood Early Resuscitation
PRBC	packed red blood cells
PROPPR	Pragmatic, Randomized Optimal Platelet and Plasma Ratios
RCT	Randomized control trial
RETIC	Reversal of Trauma-Induced Coagulopathy
ROTEM	rotational thromboelastometry
STORHM	Sang Total pour la Reanimation des Hemorragies Massives
SWAT	Shock, Whole Blood, and Assessment of TBI
TBI	traumatic brain injury
TEG	thromboelastography
THOR	Trauma Hemostasis and Oxygenation Research
TIC	trauma-induced coagulopathy
VET	viscoelastic test
WB	whole blood
WFWB	warm fresh whole blood
